# RMLNet—A Reliable Wireless Network for a Multiarea TDOA-Based Localization System

**DOI:** 10.3390/s19204374

**Published:** 2019-10-10

**Authors:** Yuan Xue, Wei Su, Dong Yang, Hongchao Wang, Weiting Zhang

**Affiliations:** National Engineering Laboratory for Next Generation Internet Interconnection Devices, Beijing Jiaotong University, Beijing 100044, China; 15111042@bjtu.edu.cn (Y.X.); wsu@bjtu.edu.cn (W.S.); dyang@bjtu.edu.cn (D.Y.); 17111018@bjtu.edu.cn (W.Z.)

**Keywords:** ultrawideband (UWB), localization, multi-area localization, medium access control (MAC), timeslot

## Abstract

Ultrawideband (UWB) wireless communication is a promising spread-spectrum technology for accurate localization among devices characterized by a low transmission power, a high rate and immunity to multipath propagation. The accurately of the clock synchronization algorithm and the time-difference-of-arrival (TDOA) localization algorithm provide precise position information of mobile nodes with centimeter-level accuracy for the UWB localization system. However, the reliability of target node localization for multi-area localization remains a subject of research. Especially for dynamic and harsh indoor environments, an effective scheme among competing target nodes for localization due to the scarcity of radio resources remains a challenge. In this paper, we present RMLNet, an approach focus on the medium access control (MAC) layer, which guarantees general localization application reliability on multi-area localization. Specifically, the design requires specific and optimized solutions for managing and coordinating multiple anchor nodes. In addition, an approach for target area determination is proposed, which can approximately determine the region of the target node by the received signal strength indication (RSSI), to support RMLNet. Furthermore, we implement the system to estimate the localization of the target node and evaluate its performance in practice. Experiments and simulations show that RMLNet can achieve localization application reliability multi-area localization with a better localization performance of competing target nodes.

## 1. Introduction

With the improvement of wireless techniques and applications, the demand for location-based services (LBS) has continuously increased in the Internet of Things (IoT) [1,2,3,4]. Self-awareness of sensor node locations is important for the network and for most applications because sensor data without spatiotemporal characteristics have limited meaning [1]. Traditional LBS is available through GNSS. However, in indoor environments, GNSS does not work well, especially in demanding communication environments. Such as in buildings, underground structures, industrial process optimization or indoor navigational aid, where weak signal conditions are encountered [5]. To solve the need for high-precision localization, the business community and groups in academia have conducted research on indoor localization technology [6,7].

**Figure 1:**
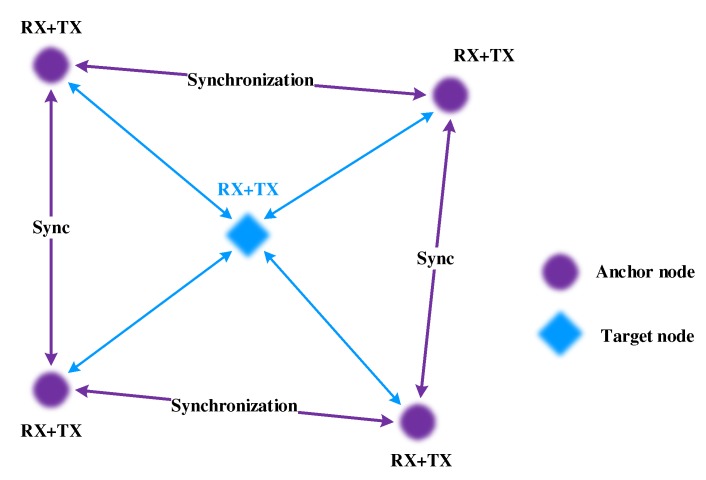
Synchronization of the time-difference-of-arrival (TDOA).

In general, the accuracy of localization based on ranging is achieved by solving a set of simultaneous equations based on distance measurement, for example, time-of-arrival (TOA) [8], time-difference-of-arrival (TDOA) [9], difference-time-difference-of-arrival (DTDOA) [10] and the received signal strength (RSS) [11]. Among these parameters, the TDOA is one of the commonly used method that has the advantages of high measurement accuracy, simultaneous removal synchronization of nodes and easy implementation and operation. In the ranging-based localization, the signal distance from the target node to the anchor nodes can be used to estimate the internode distances together with the propagation speed or the channel fading model. And then, a target node can be located by a set of anchor nodes that measure the time difference, as shown in Figure 1 [12]. The difficulty of TDOA method is to estimate the distance between nodes, especially in dynamic and industrial environments. In these environments, most narrowband signals are highly attenuated, resulting in a decrease in the ranging accuracy.

Some recent studies have shown that the placement of more anchor nodes, increased node signal transmission power [8] and enhanced cooperation between nodes can improve the accuracy of localization [9,10,11]. However, in the context of IoT, some devices have stringent energy requirements and computational requirements. Under the premise of ensuring localization accuracy, how to reduce energy consumption and extend network life to meet the localization and deployment requirements are challenges [13]. Indoor localization is gaining increasing attention and various technologies have been applied to localization including Wi-Fi, Bluetooth, radio frequency identification (RFID), acoustic signals, magnetic fields, UWB and light. Among them, due to the high ranging accuracy (<10 cm) of UWB becomes an excellent technique for precise localization. Compared with narrowband signals, UWB signals have an advantage in precise localization due to their high temporal resolution and potential for multipath resolution. However, the localization schemes commonly used with UWB have their own limitations. Due to the limited transmission distance of UWB signals, localization in multi-area network during the movement process are particularly important. In addition, the ranging and localization of fixed positions are relatively simple and the localization accuracy can be improved by continuously optimizing the error. However, localization the moving node requires not only the frequency of the localization but also requiring the transmission power of the signal, the collision loss rate and so on.

For mobile nodes, which are generally powered by batteries, resources are limited. The lifetime of location-aware network will affect the survival of the localization system. Therefore, a location-aware network requires lower power consumption [14,15,16]. The IEEE 802.15.4a standard [17], which defines several deterministic channel access schemes, is a valuable candidate for communication requirement systems in low-power-consumption networks. The standard imposes certain physical layer (PHY) guidelines, such as the transmission power. With the increasing number of anchor nodes, the reliability of localization becomes limited, as interfering messages may interrupt the required message exchange. Moreover, the localization system needs to consider the impact of the medium access control (MAC) layer protocol within practical implementations. A carrier sense multiple access (CSMA-CA), offer good benefits relating to changes within the network but are not suitable for UWB-based location-aware networks with a large of nodes due to the listen-before-talk mechanism, which requires sensing the medium. To overcome the limitations of existing approaches, the time division multiple access (TDMA) MAC approaches are more beneficial for high user densities in the location-aware network than contention-based approaches [18]. Therefore, providing satisfactory localization availability in the mobile localization process is one of the main challenges that has yet to be solved.

In a location-aware network, due to the limitation of wireless resources, only a limited number of node pairs can perform measurements between nodes simultaneously. To effectively utilize limited wireless resources (such as bandwidth) to located more nodes, the design of a targeted network transmission strategy is essential. Network transmission strategies affect location reliability. Song [19] developed a framework for evaluating metric network transmission strategies and proposed a context-aware network transmission strategy method to mitigate network localization errors. Garcia [5] established an optimization framework for a joint localization network with the goal to allocate STDMA network resources and perform UWB TW-TOA ranging. In these two localization systems, there is no system analysis of other localization aspects, such as large area coverage, tag roaming or multiuser interference, which are critical to achieving real-world indoor deployment. For example, in [14], the authors compared two existing solutions in terms of the accuracy, location update rate and end-to-end latency. In addition, the combination of TDMA and other methods, such as code division multiple access (CDMA), was studied in [9]. Although the MAC was analyzed in terms of delay, it was not shown whether this method can further improve scalability to support higher user density. In such a context, designing an effective and efficient UWB localization system is very challenging in industrial communications [20].

In this paper, we focus on the IEEE 802.15.4a standard to enable the adoption of the low-latency deterministic network (LLDN) mode as the basis for UWB location-aware networks. Furthermore, we consider the lifetime of targets with low-rate, low-power networks and the number of nodes. We present RMLNet, an efficient network addressed on the MAC layer, which guarantees the general localization application reliability on different UWB network zones. Specifically, we present how to support localization by applying slotted scheduling. The design requires specific and optimized solutions for managing and coordinating multiple anchor nodes. In addition, an approach of target area determination is proposed, which can approximately determine the region of the target node by RSSI, to support RMLNet. Furthermore, we design the system to estimate the multi-area localization of the target node and evaluate its performance. Experiments show that RMLNet can achieve localization application reliability in multi-area localization with a better accuracy of competing target nodes.

The rest of this paper is organized as follows. In Section 2, we review the studies on UWB localization networks related to our work. Section 3 describes and analyzes the overview of the TDOA-based localization system. Then, Section 4 discusses the proposed mechanism, including the proposed target area determination approach and network scheduling mechanism. The performance of our prototype network is analyzed in Section 5. Finally, the conclusions are drawn in Section 6.

## 2. Related Work

With the development of localization technology, future wireless networks will become location-aware networks that can perceive the location of the target within its deployment area. This section is divided into two parts. First, the network architectures used in localization are discussed. Then, we discuss the research on UWB localization.

### 2.1. Network Architecture

Large-scale localization systems may require a large number of anchor points to determine the bits of the mobile node. Therefore, the transmission strategy in a location-aware network is a very important topic. The existing indoor location solutions in location-aware networks can be approximately divided into two categories—distributed algorithms and centralized algorithms. In the distributed method [18,21,22,23], after each node performs a location information exchange, its information processing and calculation data are distributed throughout the network. The advantage of the distributed localization algorithm is that the system is relatively scalable and robust. In centralized localization [24,25,26], all anchor position and measurement data are forwarded to a central processor to jointly calculate unlocalized node locations. The centralized approach utilizes information about the entire network and is expected to produce more accurate location estimates. Due to the self-organizing nature of location-aware networks, distributed localization is more advantageous in implementation. However, compared with the case of centralized localization, the distributed data lack complete network data information and the localization results are not necessarily optimal. Therefore, to achieve global optimization, an effective network solution is needed to ensure large-scale data transmission and nonlinear optimization problems associated with centralized methods. For that, how to select a suitable network transmission schemes for localization in practical is a critical issue.

There are some TDMA MAC algorithms focus on UWB localization in Table 1. Tiemann et al. [27] proposed a practical TDOA system based on IEEE 802.15.4a with code division multiple approach (CDMA) for spreading the synchronization. However, the approach may may need to design a scheduled access scheme precisely tailored for multi-user application. In [28], the authors proposed a multi-cell TDMA MAC algorithms for localization specific traffic and moving nodes to request multiple slots. The roaming success rates are 93–100% at normal walking speed (1 m/s) and 89–98% at running speed (3 m/s). It can combine with [29] in a mesh network. However, it need high precision synchronization and propagate multiple synchronization messages in multi-hop deployments. Macoir et al. [30] proposed an adaptation of the Time Slotted Channel Hopping (TSCH) MAC layer design for UWB. It allows for UWB nodes to be used in low power wireless sensor networks. Moreover, it has high scalability. Our work focused on a localized network transmission, which guarantees general localization application reliability on multi-area localization.

### 2.2. Localization System

In recent years, various methods for improving localization accuracy and scalability have been extensively studied. Xue et al. [12] extended the traditional TDOA without time synchronization. Through the introduction of a reference node, time difference measurement based on one-way ranging was realized. In particular, a model was proposed and compared to the standard TDOA method. The experimental results show that the asynchronous TDOA model is superior to the standard TDOA model in terms of localization accuracy and has higher efficiency in reducing the amount of data packet transmission. However, that work did not analyze any MAC functions, nor did it study the scalability of the system. Refs. [19,31], respectively, used the TDOA and round trip time (RTMs) to reach the measurement to solve the problem of localization resource allocation in the asynchronous network. It is not possible to assess the impact of this solution on the number of supported users. Ridolf et al. [32] provides a mathematical model for calculating the theoretically supported user density of multiple localization methods in a single-domain unit (without switching) with carefully chosen settings and choices. The study addressed a single-domain, small-scale unit with a limited number of mobile nodes. In [33], a system architecture with wider coverage for more users was proposed. In this architecture, the management and control portion of the network (including the allocation of UWB resources such as time slots and device roaming) is separated from the actual localization system. However, UWB is used only for ranging and position estimation and other functions are managed by a second non-interfering Wi-Fi network.

## 3. System Model

### 3.1. System Description

**Figure 2:**
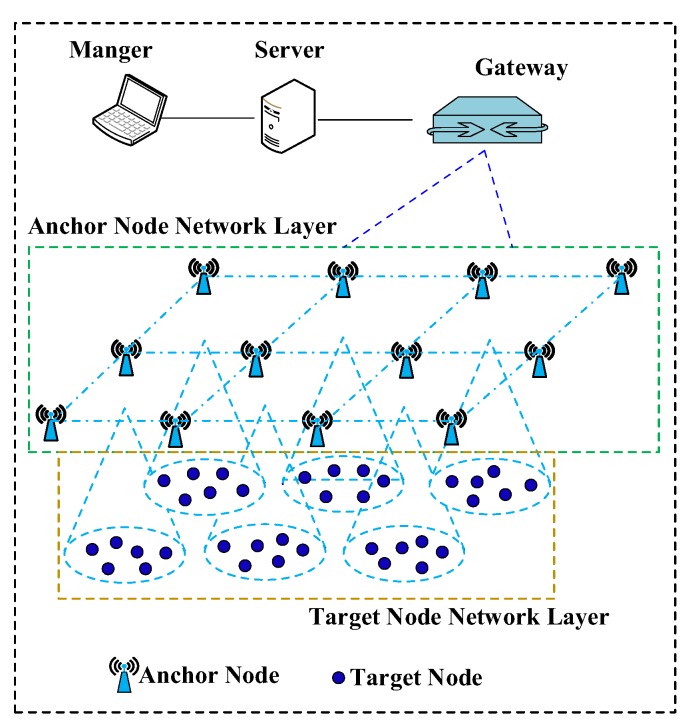
The overview of the network architecture.

The overall architecture of our current localization network is shown in Figure 2. The system consists of a manager, a gateway, several fixed anchor nodes and normal localized nodes. The manager is responsible for executing the localization algorithm and managing the wireless communication resource of the whole network. The connection between the wireless nodes and the manager is constructed by the gateway. There are two types of wireless nodes in the network—the normally localized nodes and the anchor nodes. We divide our system into two layers—the anchor node network layer (ANNL) and the target node network layer (TNNL). We use centralized management to manage the whole network since this approach can guarantee more reliable communication and maintain the real-time localization of the target nodes. The ANNL, on the one hand, is responsible for localizing the target nodes as the fixed referenced nodes. On the other hand, this layer is also responsible for collecting and sending the related information of target nodes and their own network information to the gateway and manager.

In our system, all the wireless nodes are equipped with UWB wireless technologies. IEEE 802.15.4a-2015 provides a UWB PHY layer based on impulse radio [34], which allows precision ranging and is very robust even at low transmission powers. In addition to the PHY layer, the MAC layer strongly impacts the performance of the system. The MAC layer is in charge of appropriately assigning the medium resources to the competing nodes of the system and, as such, this layer impacts the overall throughput and the channel access latencies of the system, among other parameters. Location-aware networks generally consist of a limited number of anchors with known positions and many agents with unknown positions. Proper resource allocation strategies are of great importance in typical location-aware networks for stability and reliability of the localization.

### 3.2. Localization Model

TDOA-based localization needs to measure the time difference between the target and any pair of anchor nodes to locate a target node.

Given anchor nodes $a_{1},\cdots ,a_{n}\in R^{d}$ (*d* is usually 2 or 3) and the target node $x_{S}$. The Euclidean distance $d_{sj}$ between the target node $x_{S}$ and the *j*-th anchor node for $\left(s,j\right)\in N_{a}$ satisfies $N_{a}=\{\left(s,j\right):∥x_{s}-a_{j}∥=d_{sj}\leq r\}$, where ∥·∥ denotes the L2 norm and *r* is the radio range. Thus, the transmission time $T_{si}$ between the target node $x_{s}$ and the *j*-th anchor node can be given as

(1) $$T_{sj}=d_{sj}/c=∥x_{s}-a_{i}∥/c$$

where *c* is the signal transmission speed. For the TDOA, we have clock synchronization across all receivers only and we cannot obtain the arrival times. However, we can obtain the differences in the arrival times. The TDOA $T_{ij}$ between the *i*-th anchor node and the *j*-th anchor node from the target node $x_{s}$ can be computed by

(2) $$T_{ij}=T_{si}-T_{sj}=\left(∥x_{s}-a_{i}∥-∥x_{s}-a_{j}∥\right)/c,\forall \left(a_{i},a_{j}\right)\in R^{d}$$

In TDOA-based localization, given the measurements $d_{ij}$ and anchor node positions $a_{n}$, we can estimate the localization of the target node $x_{s}$.

### 3.3. Network Model

**Figure 3:**
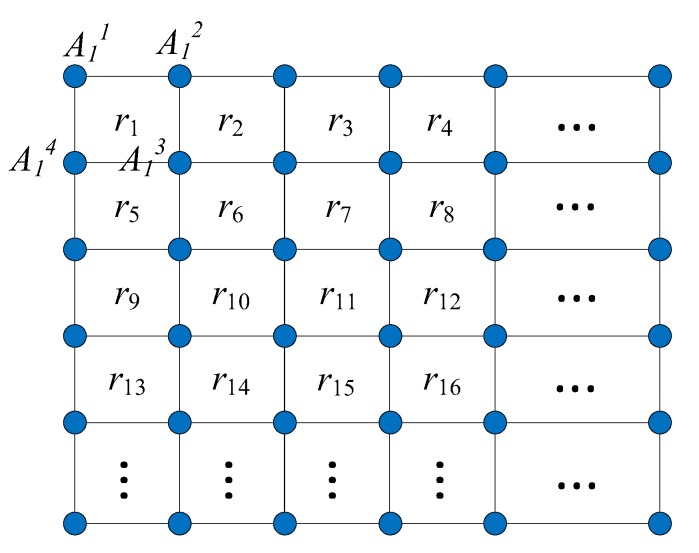
The overview of the network model.

We consider an ANNL network graph, which contains a set of anchor nodes and a limited number of target nodes. $N=\{n_{1},n_{2},\ldots ,n_{i},\ldots ,n_{\gamma }\}$ is the collection of target nodes, where γ is the maximum number of target nodes. The positions of the anchor nodes are known before the network formation. As shown in Figure 3, we use a simple such scenario to illustrate the localization system. A target node is located by 3 or 4 anchor nodes using the TDOA-based localization method. Therefore, the localization of the anchor nodes is regular andthe whole network is separated into several square areas. $R=\{r_{1},r_{2},\ldots r_{j},\ldots r_{M}\}$ represents the collection of separated target areas, where $r_{j}=\{A_{1}^{j},A_{2}^{j},A_{3}^{j},A_{4}^{j}\}$ is the collection of four anchor nodes in the *j*-th area and *M* is the maximum area among the whole network. We use $A_{k}^{j}$ to represent the *k*-th anchor node in the *j*-th area, where 1⩽j⩽M,1⩽k⩽4.

Recall that three non-collinear beacon messages are a fundamental requirement for localization and that the fully localized condition is desired. The accuracy and stability of localization are greatly affected by the reliability of message transmission, especially in industrial environments. Thus, to guarantee reliability, the scheduling-based MAC protocol, TDMA, is adopted in this paper. According to the above network model, communication resources, such as time slots, need to be scheduled for message transmission between the anchor nodes and target nodes.

## 4. System Design

This paper presents a UWB indoor localization system using a TDOA localization approach that ensure collision-free access to the network. The design requires specific and optimized solutions for managing and coordinating multiple anchor nodes. The design of a specific solution related to target area determination, anchor node slot allocation, target node intervention and movement can impact the realization of the network and the system, which is presented in this section.

### 4.1. Process of Localization

**Figure 4:**
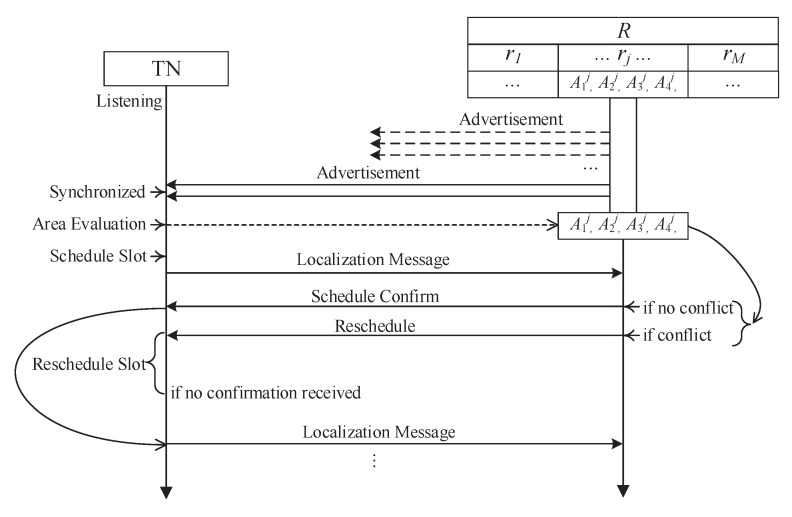
Process of localization.

The network process between the target node and anchor nodes of the localization system described in this design is shown in Figure 4. The process is as follows:

- The anchor node broadcasts a synchronization and localization signal in the public frequency band andthe target node receives it. The anchor and target complete synchronization and determine the target node region using the signal strength.
- The target node sends localization signals, including the region information, in the regional frequency band according to the regional slot scheduling strategy.
- According to the regional slot scheduling policy, the target node randomly selects the free slot in the current region and sends access requests in the access slot. If the target node does not receive the slot allocation policy in the next slot, this node considers the access request to have failed and reselects the free slot to send the access request again. The anchor node broadcasts the timeslot scheduling strategy in the regional frequency band. The target node adjusts the receiving frequency band and receives the timeslot scheduling strategy.

In the process, the phase of synchronization, area determination and timeslot schedule of the anchor node is in the broadcast state and the transmission and node access phase are in the listening state. Correspondingly, the target node has three phases in the listening state and two phases in the receiving state. The functions of each phase are described below:

- Node access—The new target node is connected to the network. The anchor node has received the access request of the new target node and issues a slot allocation strategy in the next slot as described in Section 4.2.
- Synchronization and area determination—time synchronization between the anchor and target nodes. The anchor node broadcasts the time signal in turn andthe target node approximately synchronizes its own time after receiving the time signal. The region of the target node is approximately determined. The target node receives the broadcast signal from the anchor node and determines the location according to the determination mechanism by as described in Section 4.3.
- Transmission—According to the received slot allocation strategy, the target node sends localization information to the anchor node.
- Timeslot schedule—The timeslot allocation strategy of each region is issued by the anchor node. The target node receives the slot scheduling strategy from the local region, which is sent by the anchor node and determines its own slot as described in Section 4.4.

### 4.2. Target Access

The target node moves to a new region but it does not immediately know its position, so it can determine only its access time by receiving broadcast information from the anchor node. There are two types of target node access—node startup and node movement across regions.

- Node startup—The target node is in the listening state and receives the broadcast signal from the anchor node. After completing the phases of synchronization and area determination, the target node adjusts the listening frequency to receive the scheduling signal from the anchor node in its region. The scheduling signal includes information such as the number of all target nodes in the current region and the slot allocation strategy.
- Node cross-region—The target node uses the RSSI to determine whether the cross-region is completed. If it is not completed, the transmission of localization signals is carried out according to the slot allocation strategy of the previous region. After the target node completes the cross-region movement, the target node adjusts the listening state, receives the synchronization information, localization information and scheduling information from the anchor node of the new region and randomly selects the free transmission slot in the access slot of the new region to send the access request.

That is if the target moves within the original area, it will transmit the localization signal according to the slot allocation strategy at the previous moment. If the target node moves cross-region, the target node adjusts the listening state, receives the synchronization information, localization information and scheduling information from the anchor node of the new region and randomly selects the free transmission slot in the access slot of the new region to send the access request. Note that the target node receives the slot allocation strategy in the scheduling time slot. If the target node does not receive the slot allocation information, it sends an access request in the stage of node access. We stipulate that if the anchor node does not receive the localization signal from the target node for more than three frames, which indicates that the target node has left the area or has been closed, the anchor node releases the time slot occupied by the target point in the next frame.

### 4.3. Target Area Determination

In Figure 3, the target node receives the signal from anchor nodes in different cell regions and records the RSSI. In general, *RSSI* is proportional to $d^{-\gamma }$, where *d* is the propagation distance between the target node and the anchor node and γ is the path-loss exponent. The relation between the RSSI and the propagation distance can be represented as $RSSI\propto d^{-\gamma }$ and a simplified model is given by the following:

(3) RSSI=−10γlogd+A

where *A* is Gaussian additive noise with zero mean and standard deviation $\sigma_{A}$ related to the specific connections.

**Figure 5:**
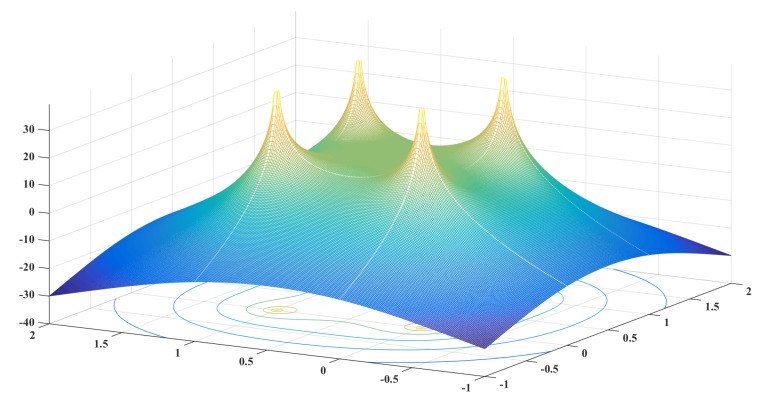
The received signal strength indication (RSSI) in one cell.

**Figure 6:**
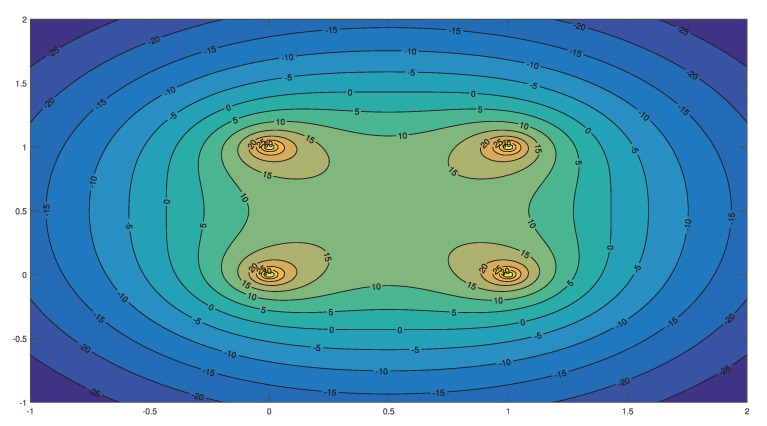
The contour lines of the RSSI.

To further describe the relationship between the RSSI measured by the target node from $A_{k}^{j}$ and the position of the target node, we tested the performance of the RSSI in a region under ideal conditions. The coordinates of anchor nodes were (0,0)(0,1)(1,0)(0,0). The results are shown in Figure 5 and Figure 6. So, in theory, the RSSI received by the target node in the region is greater than that received outside the region. Therefore, we can approximately determine the region where the target node is located by comparing the the RSSI values in different regions. We define $T_{r_{j}}$ as the region of the target to be determined, such that

(4) $$T_{r_{j}}=\arg\max_{r_{j}\in R}\{\sum_{k\in r_{j}}s_{k}^{j}\}$$

where $S_{r_{j}}=\sum_{k=1}^{4}s_{k}^{j}$ represents the RSSI value of an advertisement broadcasted by $A_{k}^{j}$, and $S_{r_{j}}$ represents the sum RSSI of node the *n* in region j. According to Equation (3),

(5) $$s_{k}^{j}=-10\gamma \log d_{k}^{j}+A$$

We can approximately determine the region of the target node by Equation (4). The region with the maximum is the region where the node $T_{r_{j}}$ is located.

Figure 5 and Figure 6 show that $\sum_{k=1}^{4}s_{k}^{j}$ is generally larger near the anchor node in region j. Theoretically, the RSSI has an extreme value in the region far from the anchor node and a maximum value at the regional center. The RSSI decreases along the regional center to the midpoint direction of the anchor nodes and reaches the minimum value at the midpoint. Thus,

**Theorem** **1.** *If there is a maximum value of the RSSI at the regional center, then the minimum value of the RSSI within the region is at the midpoint of the regional boundary.*

Thus, there is a minimum value of the RSSI in the region near the midpoint of the anchor nodes. All values in this region should be greater than the minimum value. Thus, we can introduce a threshold determination mechanism to locate the region of the target node more accurately. As mentioned above, $\sum_{k=1}^{4}s_{k}^{j}$ is mainly affected by the nearby anchor node and changes little near the boundary of the region, which affects the region determination of the target node. We introduce $S_{threshold}$, the minimum value of the RSSI in the region as the threshold determination to assist in locating the region of the target node.

(6) $$T_{rj}=\underset{r_{j}\in R}{\arg\max}\left\{\mathrm{sign}\left(S_{r_{j}}-S_{threshold}^{r_{j}}\right)\right\}$$

where $S_{threshold}^{r_{j}}$ represents the minimum value of the RSSI in the region $r_{j}$.

We can approximately determine the region of the target node by Equation (6), as the target node communicates near the boundary of the region. However, due to signal transmission errors, multiple values $T_{r_{j}}$ close to each other can be obtained by Equation (6). At this point, the region where the target node is located needs to be further determined.

The region where the target node is located depends on the anchor node, which is farthest from the target node among the four anchor points constituting the region. For cases where multiple values are similar, we can calculate the minimum of $s_{k}^{j}$ in each region and then obtain the maximum value of these minimum values.

(7) $$T_{r_{j}}=\underset{r_{j}\in R}{\arg\max}\{\min_{m\in r_{j}}\left(S_{m}\right)\}$$

$S_{m}$ represents a collection of RSSI values measured by the target node m in all regions.

The region containing the anchor node with the largest RSSI is the region containing the target node. Therefore, we can determine the region of the target node by Equation (7) when there are multiple values $r_{j}$ close to each other as obtained by Equation (6).

Thus, we can determine the region of the target node by Algorithm 1.

 **Algorithm 1:** Determine the region

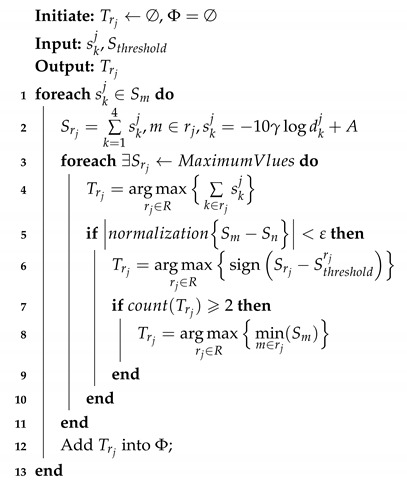



### 4.4. Slot Scheduling

The ANNL adopts the TDMA MAC protocol for all communication. We give the communication resources scheduling in this subsection according to the scenario displayed in Figure 3 and the overview. The process of localization is demonstrated in Figure 4. First, when a target node first moves into a new target area, the node needs to be synchronized by the advertisements broadcast by the anchor nodes. Through such advertisements, the target node can evaluate its area and preschedule the timeslot for the transmission of localization messages. Second, the target node should send the localization-related information to anchor nodes after synchronization. Then, the anchor nodes send the scheduling information to the target nodes. The scheduling is confirmed when the target node received confirmation. A conflict may occur when more than two target nodes move into the same target area at the same time. Thus, we schedule the final step downstream to avoid the resource conflicts and improve the reliability of communication resource scheduling.

**Figure 7:**
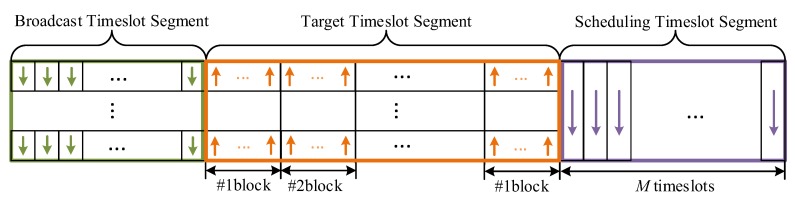
Construction of a superframe.

Several repeated timeslots construct a superframe. As shown in Figure 7, we divide a superframe into three segments—the broadcast timeslot segment (BTS), the target timeslot segment (TTS) and the scheduling timeslot segment (STS). In the design, a superframe contains of 500 timeslots, each of the timeslot is 20 ms. The setting of the timeslot mainly considers two factors—the transmission rate of the radio chip and the time during the server calculates the localization result. First, the transmission rate of data packets in the RF chip is 6.8 Mbps and the size of each packet is 127 Bytes. In theory, the time it takes to send a packet is 0.15 ms. Second, the server to solve a set of coordinate values using the TDOA algorithm is less than 10 ms.

**Figure 8:**
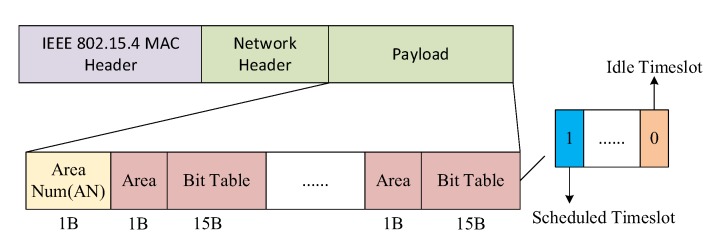
Construction of the advertisement packet.

In the BTS, each anchor node broadcasts in its own area. The advertisement packet contains the timeslot bit table. That is, each anchor node announces the current timeslot scheduling in its own area among TTSs. The designed format of the advertisement packet is shown in Figure 8. The advertisement packet contains an IEEE 802.15.4 MAC header, network header and payload. We use the payload mainly to tell the target node the current usage of timeslots. The payload consists of the number of areas (AN) and the serial number of areas and their bit table. One of the bits in the bit table, ‘0,’ represents unscheduled timeslots in a block and‘1’ represents scheduled timeslots. Each bit table represents one block of timeslots in the TTS. Once the target node receives the advertisement packets, it disassembles the area and bit table. According to the area self-evaluated in Algorithm 1, the target randomly chooses an idle timeslot in the corresponding bit table as its upstream timeslot in the TTS.

The TTSs supply localized information of the target node. Since the target areas are different, each area needs a block of timeslots. To save resources, different areas can reuse the same timeslots. Thus, we design Algorithm 2 to generate a block of timeslots. Ω represents the temporary collection for storing the scheduled area and $B=\{b_{1},b_{2}\ldots \}$ is a collection of timeslot blocks. We adopt the graph coloring algorithm to solve the allocation of upstream timeslots. Each block contains several timeslots to support the target node in an area. The number of timeslots of a block depends on the maximum number of target nodes in an area. The adjacent area cannot use the same block because that condition would result in wireless interference and the sharing of same two anchor nodes. However, different areas can use the same block if they do not share the same anchor nodes. Based on our experience, we assume that no wireless interference occurs if the areas are not adjacent.

 **Algorithm 2:** Schedule

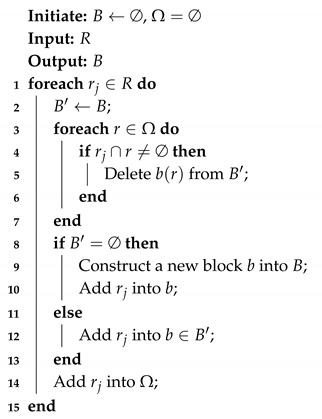



The STSs are used downstream for scheduling information. In a block, a timeslot can be used for one target node only. Thus, if more than one target node selects the same timeslot in the same area, a conflict may arise. When the anchor nodes receive the localization packet of a target node, they send the scheduled information to the target node in the corresponding timeslot. This packet is multicast. Thus, when the other target nodes receive the packet, they will find that they are not scheduled. Thus, they will reselect the upstream timeslot in the next superframe. Each area selects a major anchor node to send this scheduling information.

## 5. Implementation and Experiments

In this section, we first introduce the system based on RMLNet and then evaluate the performance of the stability and accuracy of the RMLNet-based localization system.

### 5.1. System Implementation

To evaluate our proposed RMLNet, we designed a system based on UWB, which can overhear signals and mark the recorded messages with a timestamp. We used the DecaWave DW1000 as radio transceivers, which is compliant with the IEEE 802.15.4-2011 standard. The controlling framework of transmitting or receiving the timestamp was actualized by an STM32F105 chip with Contex-M3. We integrated these nodes into the RMLNet network.

In this system, target nodes are arbitrarily deployed in the sensing field and each node has the same transmission range and similar hardware configurations. The whole sensing field is partitioned into grids. As discussed in the previous sections, the RSSI is utilized to localize the area of the target nodes first. Then, the target node sends localization signals in the regional according to the regional slot scheduling strategy. We measure the TDOA by ASync-TDOA [12]—a model for TDOA localization without time synchronization—which achieves the time difference in a one-way-based range by introducing the reference node. After calculating the TDOA, the localization of the target node is estimated by the optimization algorithm.

### 5.2. Experimental Scenario

**Figure 9:**
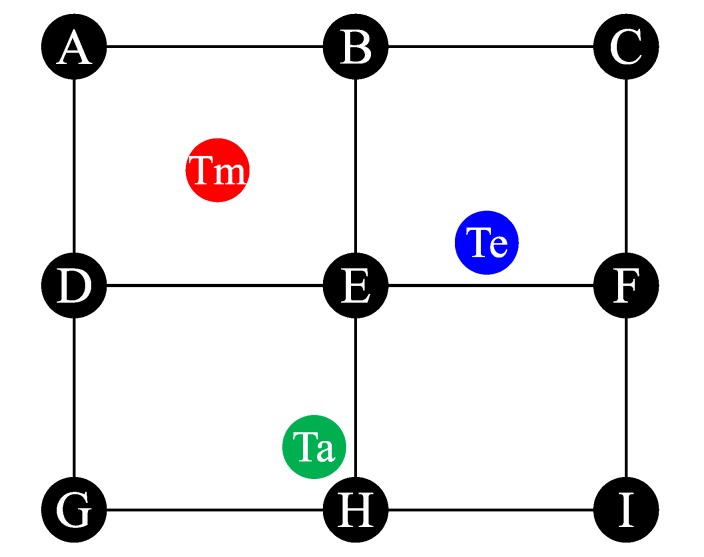
Experimental scenario.

We performed extensive experiments to examine the feasibility of ASync-TDOA for localization in a large-scale wireless network. As shown in Figure 9, we used a laboratory to simulate the factory environment. For simplicity, we considered a 2D localization model to compare with other models. We measured the time difference and estimated the localization in this environment to evaluate the proposed model.

The coordinates of anchor nodes are $A\left(A_{1}^{1}\right)=\left(0,4.8\;m\right)$, $B\left(A_{1}^{2}/A_{2}^{1}\right)=\left(2.4\;m,4.8\;m\right)$, $C\left(A_{2}^{2}\right)=\left(4.8\;m,4.8\;m\right)$, $D\left(A_{1}^{4}/A_{3}^{1}\right)=\left(0,2.4\;m\right)$, $E\left(A_{1}^{3}/A_{2}^{4}/A_{3}^{2}/A_{4}^{1}\right)=\left(2.4\;m,2.4\;m\right)$, $F\left(A_{2}^{3}/A_{4}^{2}\right)=\left(4.8\;m,2.4\;m\right)$, $G\left(A_{3}^{4}\right)=\left(0,0\right)$, $H\left(A_{3}^{4}/A_{4}^{4}\right)=\left(2.4\;m,0\right)$ and $I\left(A_{4}^{3}\right)=\left(4.8\;m,0\right)$. We defined three target scenes—near the middle $\left(T_{m}\right)$, near the anchor node $\left(T_{a}\right)$ and near the edge of a line between two anchors $\left(T_{e}\right)$. In all experiments, the target node is static and transmits a packet every 200 ms. The localization of each setting was conducted many times.

### 5.3. Target Area Determination

**Figure 10:**
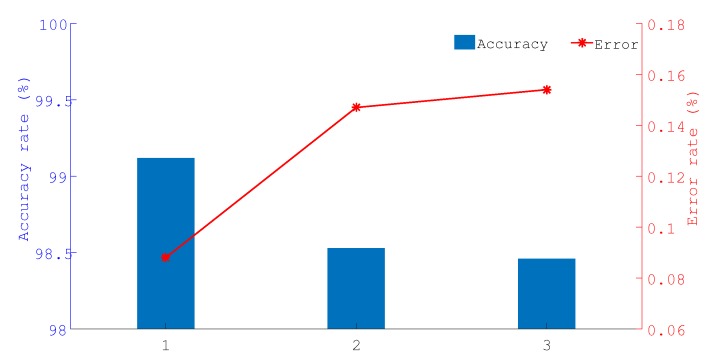
The accuracy rate of target area determination.

This test mainly evaluated the performance of the target area determination. As shown in Figure 9, 4 target nodes are placed in each scene at $T_{m}$, $T_{a}$ and $T_{e}$, and each test is conducted 1000 times. Figure 10 shows the percentage ratio of the correct times to the test times. Here, ‘1’ represents the scene of the target being near the middle ‘2’ represents the scene of the target being near the anchor node and‘3’ represents the scene of the target being near the edge of a line between two anchors. As shown in Figure 10, the proposed project of the determine region has a better success rate, which can exceed 98.5% and guarantee the performance of the network for multi-area localization.

### 5.4. Localization Performance

This test compares the performance of the localization in the RMLNet network and CSMA network with the results for the ASync-TDOA and traditional TDOA, respectively. As shown in Figure 9, 12/14/16/18/20 nodes are placed in each region and a total of 48/56/64/72/80 nodes are placed in each scene.

**Figure 11:**
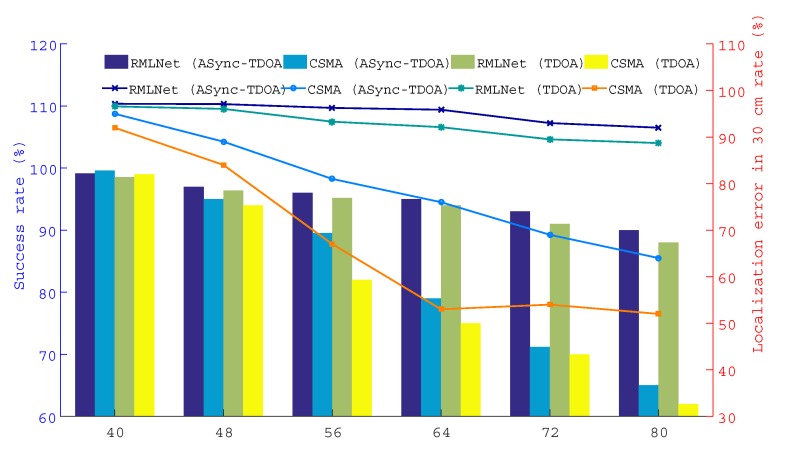
The performance of the localization test when the target is positioned near the middle.

**Figure 12:**
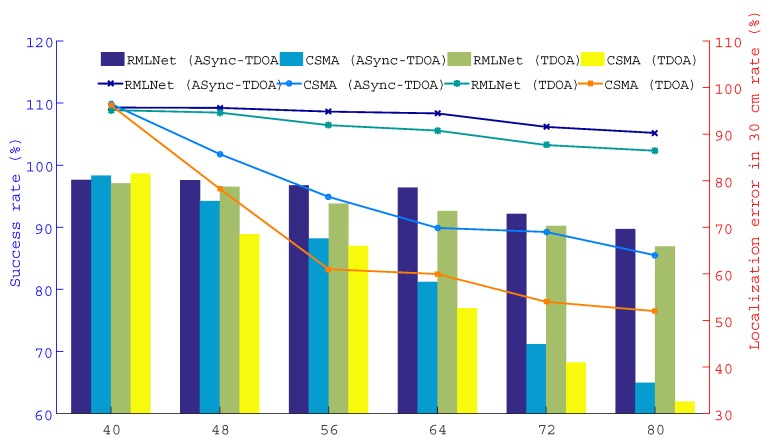
The performance of the localization test when the target is positioned near the anchor node.

**Figure 13:**
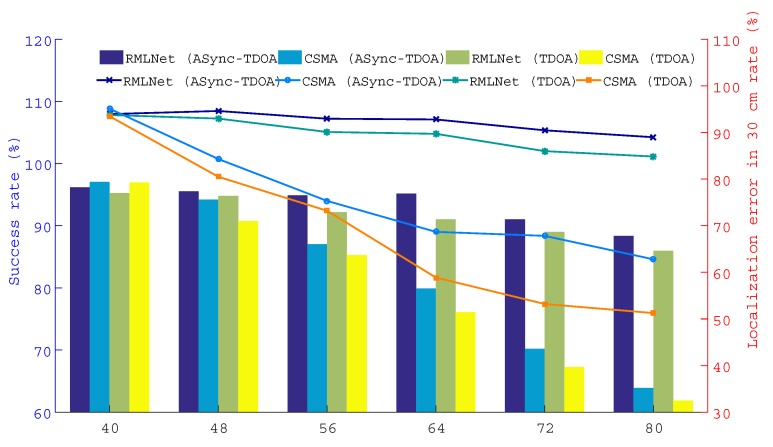
The performance of the localization test when the target is positioned near the edge of a line between two anchors.

The results are shown in Figure 11, Figure 12 and Figure 13, which show the successful localization of the RMLNet network and CSMA network based on the ASync-TDOA or traditional TDOA and the percentage of successful localization errors within the range of 30 cm. The bar graph represents the percentage of successful localization events versus the total test number, while the broken line graph represents the percentage of localization errors within the range of 30 cm. Figure 11 shows the results for the target nodes being near the center, Figure 12 shows the results for the target nodes being near the anchor point and Figure 13 shows the results for the target nodes being near the boundary. We can see that the location effect of the RMLNet network is better than that of the CSMA network using two TDOA methods.

There are 40 target nodes for localization, the location results of the RMLNet-based localization are worse than those of the CSMA-based localization. The reason is that few target nodes and less competition in the CSMA network, that will not cause data conflicts. However, the process of localization in the RMLNet network needs to execute regional evaluations, which may fail and affect the performance of localization. At this time, the interference that affect the wireless transmission mainly comes from the NLOS and the electromagnetic operation of the device, which may cause the link transmission to fail. And the success rate of the localization cannot reach 100%. With the increases of the number of target nodes, the success rate of localization based on both networks are decreased. But the performance of RMLNet-based localization is still better than that of CSMA-based. The reason is that when target nodes are located in the same area, the more target nodes there are, the more likely transmission collisions are. The transmission of CSMA-based network is based on the competition mechanism, which decreases the success rate of the localization. The RMLNet network has designed a time slot allocation strategy, which basically guarantees the reliability of localization information transmission. However, due to interference between more target nodes, the success rate of localization will be decreased.

Furthermore, the probability of error of the RMLNet-based localization at 30 cm is greater than that of CSMA-based. With additional nodes, the localization performance based on the CSMA network is worse than that of the RMLNet network because the RMLNet network localization process does not conflict and because the success rate of localization is high. For the same localization model, the data acquired by the network with good performance has high reliability, which leads to more localization events. Moreover, the stable network performance lays a foundation for the accurate measurement of data.

In addition, as shown in the Figure 11, Figure 12 and Figure 13, ASync-TDOA localization performs better in both networks than traditional TDOA localization, especially in CSMA-based networks. The reason is that the ASync-TDOA location does not require time synchronization and involves less communication between nodes. The traditional TDOA can be located only after synchronization but the synchronization results affect the localization. As synchronization fails, the success rate of localization is affected.

**Figure 14:**
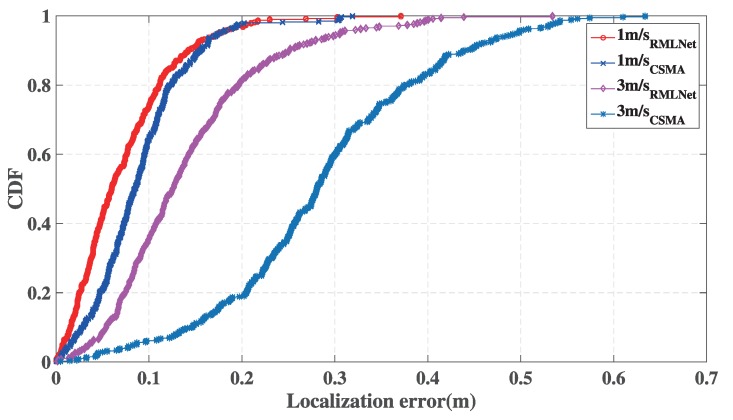
The performance of the localization at different speeds.

Figure 14 shows the localization performance at different speeds. We set 10 target nodes to move randomly for 10 min at different speeds in the scene shown in Figure 9 at 1 m/s and 3 m/s. The CDF of the statistical localization error of the test node in different networks is calculated by the ASync-TDOA [12].

Overall, the performance of the RMLNet-based localization is better than CSMA-based localization. With the increase of the moving speed, the error of the localization increases. The reason is that the nodes in the network are waiting for a response will take some time and the localization will change during that time. When the speed of the target node is slowly, the node moves at a small distance or does not move and the performance of the network can ensure that nodes obtain comparatively accurate information for localization. Increasing the speed, the localization of the target node changes rapidly, which causes the measured localization information are changing and the error of localization is increasing. When the target node moves at 1 m/s, the localization error of the RMLNet-based localization is within 0.3 m for 99% and that of CSMA-based localization is within 0.3 m for 98.6%. When the target node moves at 3 m/s, the localization error of the RMLNet-based localization is within 0.4 m, while that of the CSMA-based localization is within 0.65 m. The RMLNet network reduces collisions and ensures the reliability of each localization. During the process of the cross-region, the location information of the node is obtained with a large error or missing andthe localization error is increasing or cannot be located. The localization based on the RMLNet network incorporates a mechanism of target area determination, which restricts the target nodes in the area. Use of the RMLNet network improves the success rate and accuracy of localization.

**Figure 15:**
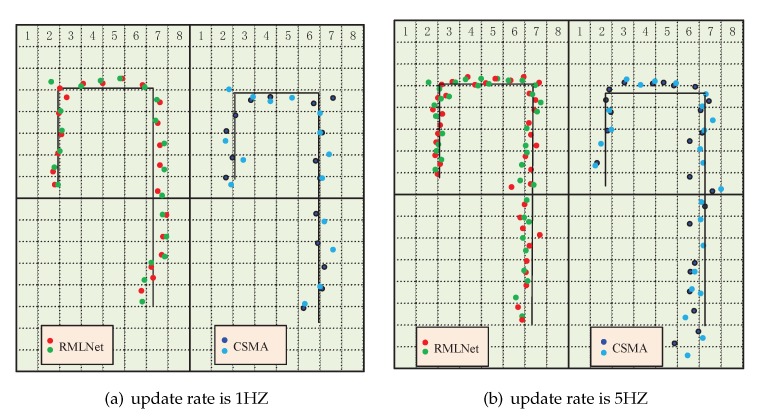
The location trajectory of two target nodes in different networks.

Figure 15 shows the location trajectory of two target nodes in different networks. We can see that the update rate of the target node will make a difference results in TDOA localization. For the CSMA-based localization, when the update rate of the target node is 1 HZ, the target will occasionally be lost and drifted. When the update rate of the target node is 5 HZ, the trajectory will have obvious discontinuities and the target cannot be located. For the RMLNet-based localization, when the update rate of the target node is 1 HZ, the location trajectory of two target nodes are stability. When the update rate of the target node is 5 HZ, the target will occasionally be lost and drifted. The reason is that the increase of the update rate will inevitably increase the communication load and the probability of collision of the data packet increases. If there is a collision between data transmissions, high-reliability channel access cannot be ensured by CSMA-based network, which is based on the competition mechanism. The RMLNet network has designed a time slot allocation strategy, which basically guarantees the reliability of localization information transmission. However, due to the interference of target nodes, the location trajectory of two target nodes will occasionally be lost and drifted.

Based on the above results, we can conclude that, first, the region of the target node can be approximately determined by the proposed RMLNet. Second, during the switching of the localization area in the movement process, RMLNet achieves reliable localization on multi-area with a better accuracy and stability of competing target nodes.

## 6. Conclusions

In this paper, we presented the RMLNet wireless network for a multi-area TDOA-based localization system to guarantee reliable switching of the localization area in the movement process. In the case of motion localization, not only to requiring accurate position information but also to improve the reliability and stability of the localization. Through research on the existing literature, we investigated the feasibilities of RMLNet wireless networks for UWB systems to improve the reliability and stability of the localization. The results showed that RMLNet can approximately determine the region of the target node by the RSSI. During the process of localization in multiple-areas, RMLNet can provide a reliable network for localization transmission with active nodes and obtain more localization information to improve the localization precision.

## Figures and Tables

**Table 1:** Summary of related works.

Ref.	Objective	MAC	Technologies	Main Contribution	Results
[27]	Higher update rates; Lower power consumption	802.15.4 CDMA	UWB	A novel method to ensure a stable clock synchronization under high system loads.	The receive ratio for synchronization frames may be held at at least 90%.
[29]	Improve the coverage and scalability	802.11 TDMA	UWB+WIFI	A TDMA MAC protocol that combines WiFi and UWB technologies.	Up to 100 client tags can request their own location update interval and corresponding UWB ranging slots are provided accordingly. Roaming between different anchor points is supported andduring the roaming procedure, communication and location updates are interrupted for at most 150 ms in a 4-hop network.
[28]	Localization specific traffic; Ensures fair and collision-free	802.15.4 TDMA	UWB	Design a multi-cell MAC protocol and management algorithms to cope with challenges such as multi-cell slot allocations, cell handovers and resource re-usage.	The scalability to 88.3% effective spectrum usage, mobile nodes are able to roam successfully in 90% of the handovers.
[30]	Higher channel efficiency; Deterministic delays; Increased robustness	802.15.4 TDMA	UWB	Combine TDMA with frequency hopping.	Allow to reach a Packet Delivery Ratio higher than 99.999% at a slot rate of 400 slots per second (time slot duration is 2.5 ms).
